# Antibacterial Activity of Bacteriocin-Like Inhibitory Substances (BLIS) of *Levilactobacillus brevis* PG-11 against *Salmonella* Typhimurium

**DOI:** 10.4014/jmb.2502.02011

**Published:** 2025-07-14

**Authors:** Guo Pan, Xinling Zhao, Changjiang Wang, Yezhengzheng Tian, Jingyu Gao, Wanli Sha, Baishuang Yin, Wenlong Dong

**Affiliations:** 1 College of Animal Science and Technology, Jilin Agricultural Science and Technology University, 132000 Jilin, P.R. China; 2 Jilin Provincial Key Laboratory of Preventive Veterinary Medicine, 132000 Jilin, P.R. China; 3 Jilin Province Technology Innovation Center of Pig Ecological Breeding and Disease Prevention and Control, 132000 Jilin, P.R. China; 4 Jilin Province Cross Regional Cooperation Technology Innovation Center of Porcine Main Disease Prevention and Control, 132000 Jilin, P.R. China

**Keywords:** Antimicrobial, bacteriocin, antibacterial mechanism, *Levilactobacillus brevis*, *Salmonella* Typhimurium

## Abstract

*Salmonella* Typhimurium is a major cause of foodborne illness. This study investigated the antibacterial activity, characterization, and mode of action of bacteriocin-like inhibitory substances (BLIS) produced by *Levilactobacillus brevis* PG-11, isolated from traditional Chinese kimchi. The BLIS of *L. brevis* PG-11 exhibited strong antibacterial activity against *S*. Typhimurium ATCC 14028. It also exhibited broad-spectrum antimicrobial activity against various foodborne and human pathogens, including *Escherichia coli*, *Staphylococcus aureus*, *Klebsiella pneumoniae*, *Pseudomonas aeruginosa*, and *Staphylococcus epidermidis*. The minimum inhibitory concentration (MIC) of the BLIS against *E. coli*, *S*. Typhimurium, and *S. aureus* was 62.5, 125, and 125 mg/ml. The active antibacterial substances were partially purified using dialysis and tricine-SDS-PAGE, revealing a molecular weight of less than 1 kDa for the active antibacterial substances. Furthermore, the BLIS was stable under acidic pH (pH 3.0-6.0), heat treatment (121°C, 20 min), UV radiation (4 h), and sensitivity to papain. Maximum antibacterial activity was observed during the stationary growth phase after 32 h of culture. Propidium iodide (PI) uptake, ATP leakage, and scanning electron microscopy (SEM) confirmed that BLIS disrupted cell membrane integrity, leading to cell membrane damage pore formation, and bacterial growth inhibition. These results highlight the potential of *L. brevis* PG-11 BLIS as a natural biological preservative for food preservation applications.

## Introduction

*Salmonella* Typhimurium is a Gram-negative, facultative intracellular bacterium responsible for systemic infections and gastroenteritis [1]. As a common foodborne pathogen, *S*. Typhimurium causes diarrheal diseases and is primarily transmitted through contaminated food or water [2]. Unlike many other foodborne pathogens, it has a broad host range, including mammals and reptiles. Its presence in food animals, such as poultry, pigs, and dairy cattle, increases the risk of transmission to humans via food consumption [3, 4]. Traditional sources of *S*. Typhimurium outbreaks include poultry, eggs, pork, and dairy products. However, recent studies have also linked contamination of fresh produce with this pathogen [3, 5, 6]. Over the past decade, between 87,000 and 135,000 human cases of salmonellosis are reported annually in Europe alone [7]. This underscores the urgent need for effective control measures to mitigate the transmission of *S*. Typhimurium and reduce foodborne outbreaks globally.

In recent years, there has been growing concern about food safety as it may cause allergic reactions and promote the formation of carcinogenic compounds. Furthermore, the overuse of antibiotics has contributed to the emergence of multidrug-resistant bacteria [8, 9]. Consequently, natural food preservation methods, particularly bacteriocins produced by lactic acid bacteria (LAB), have garnered significant interest as safe and effective alternatives to chemical preservatives [10, 11]. Bacteriocins are ribosomally synthesized antibacterial peptides or proteins that exhibit a broad spectrum of antimicrobial activity [11, 12]. LAB and their metabolites, including bacteriocins, are generally recognized as safe (GRAS) for use in food preservation due to their natural occurrence in the human gut, historical use in fermented foods, and absence of harmful side effects [13, 14]. Bacteriocins such as nisin and pediocin have been widely applied in the food industry, showcasing their non-toxic, non-allergenic, and non-mutagenic properties [15]. Moreover, LAB bacteriocins specifically target pathogenic bacteria without harming beneficial microbiota or human cells [16]. Additional functions of LAB bacteriocins include biofilm inhibition, antiviral activity, and anticancer effects [17]. Bacteriocins produced by LAB exhibit desirable properties, such as inactivation by proteases, tolerance to broad pH ranges, high heat resistance, and a relatively broad antimicrobial spectrum [10, 18]. Despite the success of bacteriocins like nisin in the food industry, their antibacterial activity is primarily limited to Gram-positive bacteria, with minimal effectiveness against Gram-negative pathogens [19]. Thus, the development of novel bacteriocins with broader-spectrum activity is essential [20, 21].

Although LAB bacteriocins have been extensively studied, relatively little research has focused on *L. brevis* [22]. Historically, bacteriocins produced by *L. brevis* 27 were effective against *Bacillus stearothermophilus*, a common spoilage organism in canned foods, but showed no activity against Gram-negative bacteria [23]. A study has demonstrated the antimicrobial activity of *L. brevis* FPTLB3 against *Escherichia coli* and *S. aureus*, with its bacteriocin exhibiting a molecular weight of approximately 54 kDa [22]. Additionally, *L. brevis* DF01 was found to strongly inhibit biofilm formation by *E. coli* and *S*. Typhimurium [21]. Recently, a novel edible coating combining the cell-free supernatant of *L. brevis* G145 and Shirazi balangu seed mucilage improved the sensory acceptability and shelf life of lamb meat during cold storage [24]. These findings highlight the potential of *L. brevis* bacteriocins for application in the food industry. Although many studies have investigated the antimicrobial properties of various bacteria, data on the antimicrobial activity of *L. brevis* against *S*. Typhimurium remain limited. Furthermore, the beneficial properties of *L. brevis* in traditional Chinese kimchi have yet to be fully explored. *L. brevis* PG-11, isolated from traditional kimchi vegetables in Yanji, China, is a relatively safe source of probiotics and demonstrates excellent antibacterial activity against *S*. Typhimurium. Research on *L. brevis* PG-11, particularly its production of bacteriocin-like inhibitory substances (BLIS), is important for enhancing the understanding of the probiotic potential of lactic acid bacteria in traditional Chinese kimchi.

This study evaluates the antibacterial activity of BLIS produced by *L. brevis* PG-11 against *S*. Typhimurium ATCC 14028 and investigates the biological properties of BLIS. Furthermore, the inhibitory mechanism of BLIS was investigated using growth inhibition assays, propidium iodide uptake (PI) analysis, ATP leakage and scanning electron microscopy (SEM).

## Materials and Methods

### Strains and Growth Conditions

*L. brevis* PG-11 was originally isolated from traditional fermented kimchi from Yanji, China. The samples were serially diluted and inoculated into MRS medium (Hopobio, China) for 24 h, 37°C. A single colony was selected, repeatedly streaked for purity and confirmed to be homogeneous before being stored at -80°C in 25% (v/v) glycerol. The 16S rRNA gene sequence of *L. brevis* PG-11 has been deposited in GenBank under the accession number PQ047826. For subsequent experiments, *L. brevis* PG-11 was cultured in MRS broth at 37°C for 24 h with 2% (v/v) inoculum. *S*. Typhimurium ATCC 14028, *S*. Typhimurium SH107, *E. coli* ATCC 25922, *S. aureus* ATCC 25923, and *K. pneumoniae* TY-30 were grown in LB broth (Hopobio, China) at 37°C for 12 h. Additionally, *P. aeruginosa* Z05, *Acinetobacter baumannii* ZXL-113 and *S. epidermidis* MR-75 were cultivated in BHI broth (Hopobio) at 37 °C for 24 h.

### Antimicrobial Activity Measurement

*S*. Typhimurium ATCC 14028 was harvested by centrifugation at 7,378 ×*g* for 5 min, washed three times with sterile saline solution (0.85% NaCl), and resuspended. The bacterial concentration was then adjusted to 1 × 10^6^ CFU/ml [25]. *L. brevis* PG-11 was cultured in 300 ml MRS broth at 37°C with continuous shaking at 120 rpm for 48 h. After incubation, the culture was centrifuged at 3,625 ×*g* for 15 min using a TGL16M centrifuge (Yancheng Kate, China). The supernatant was subsequently filtered through a 0.22 μm polyethersulfone millipore membrane to obtain the cell-free supernatant (CFS). To neutralize the effect of organic acids, the pH of CFS was adjusted to 5.0 with 2N NaOH before filtration [26]. Catalase was added at 1 mg/ml to test for residual antimicrobial activity, while untreated CFS served as a control. Antimicrobial activity was assessed using the Oxford cup agar-well diffusion assay [27]. *S*. Typhimurium ATCC 14028 suspension (0.1 ml, 1 × 10^6^ CFU/ml) was spread onto MH agar plates. Agar wells were prepared using 8 mm Oxford cups, into which 0.1 ml of CFS was added. The plates were allowed to diffuse for 1 h at room temperature, followed by incubation at 37°C for 12 h. The diameter of the inhibition zones was measured using a Vernier caliper.

### Determination of Minimum Inhibitory Concentration (MIC) and Antibacterial Spectrum

The MIC assay was slightly modified as described by Rahmati-Joneidabad *et al*. [28] PG-11 CFS was lyophilized and dissolved in sterile water to a final concentration of 500 mg/ml (w/v). The Indicator bacteria were cultured according to the method in 2.1 above and diluted to 1 × 10^6^ CFU/ml. The standard broth microdilution method was used to evaluate the minimum inhibitory concentration of *L. brevis* PG-11 CFS against pathogenic microorganisms. MIC was determined as the lowest concentration that effectively inhibited the visible growth of pathogenic microorganisms [29]. The Nisin Z (Yuanye, China), was used as a positive control. The culture conditions of indicator bacteria are as described in Strains and Growth Conditions part.

### Estimation of Molecular Mass

*L. brevis* PG-11 was inoculated into 1,200 ml of MRS broth with 2% (v/v) and cultured at 37°C for 48 h. The concentrated CFS was then dialyzed in 1.5 L of distilled water using a dialysis membrane with a molecular weight cut-off of 1,000 Da (Boyotime, China) at 4°C for 24 h. Following dialysis, the solution was concentrated 10-fold again at 45°C to obtain the crude bacteriocin extract [30, 31]. The antimicrobial activities of both the retentate and the dialysate were evaluated.

The BLIS produced by *L. brevis* PG-11 is considered a low-molecular-weight protein and was analyzed using the tricine-SDS-PAGE method, which is commonly used for proteins ranging from 1 to 100 kDa in molecular mass. Molecular weight analysis was performed as described by Schagger [32] using a vertical slab gel apparatus (Mini-Protean II, Bio-Rad). The stacking gel contained 4% acrylamide, the spacer gel 10%, and the separation gel 18%acrylamide. A molecular weight marker comprising peptides ranging from 2.7 to 40 kDa (Sangon, China) was used as a standard. The electrophoresis process began with a voltage of 30 V for 10 min, followed by 80 V until the spacer gel was completely formed, and completed at 120 V. After electrophoresis, the gel was fixed for 30 min in a solution containing 50% methanol and 10% acetic acid, rinsed with sterile distilled water, and stained with Coomassie Brilliant Blue R-250 to visualize protein bands. Additionally, antibacterial activity was tested using a gel prepared under the same electrophoretic conditions, as described by Cloeckaert *et al*. [33]. After electrophoresis, the gel was rinsed three times with sterile water, placed on a clean plate, and overlaid with MH agar containing *S*. Typhimurium ATCC 14028 (1 × 10^6^ CFU/ml). The plate was incubated at 37°C for 12 h, and zones of inhibition were examined.

### pH, Heat, Proteases and UV Rays Tolerance

The CFS was incubated at pH values of 3.0, 4.0, 5.0, 6.0, 7.0, 8.0, 9.0, and 10.0 for 1 h. MRS broth adjusted to the corresponding pH levels was used as a control. The CFS was exposed to temperatures of 50°C, 80°C, 90°C, and 100°C for 1 h, and to 121°C for 20 min to evaluate thermal stability. The pH of the CFS was adjusted to the optimal range for each protease (proteinase K, pronase E, pepsin, papain, and trypsin) using 2N NaOH or 1N lactic acid. Proteases were added to the CFS at a final concentration of 1 mg/ml and incubated at 37°C for 2 h. Proteases were subsequently inactivated by heating the samples in a water bath at 100°C for 10 min after which the pH was readjusted to the initial value of the CFS (pH 5.0). The CFS was exposed to UV radiation for 0.5, 1, 2, 3, and 4 h. Untreated CFS (pH 5.0) was used as the control group. The impact of these treatments on the antibacterial activity of the CFS was assessed using the agar-well diffusion assay [34-36].

### BLIS Production Kinetics

To optimize culture conditions, the production kinetics of BLIS by *L. brevis* PG-11 was evaluated following the method of Somashekaraiah *et al*. [37] with minor modifications. *L. brevis* PG-11 was cultured with a 2% inoculum in MRS broth at 37°C for 36 h. Samples were collected at two-hour intervals to monitor pH changes and optical density at 600 nm. The cultures were then centrifuged at 7,378 ×*g* and 4°C for 5 min (Sigma, Germany). The antimicrobial activity of the filtered supernatant was determined using the agar-well diffusion assay.

### The Growth Curves of *S*. Typhimurium ATCC14028

The effects of the CFS from *L. brevis* PG-11 on the growth of *S*. Typhimurium ATCC 14028 were evaluated following the methods of Yan *et al*. [38] and Wayah and Philip [8], with slight modifications. Briefly, *S*. Typhimurium ATCC 14028 was cultured to the exponential growth phase at 37°C. The bacterial culture was centrifuged at 7,378 ×*g* and 4°C for 5 min, washed three times with sterile saline, and OD_600_ was adjusted to 0.6. Subsequently, 10 ml of *L. brevis* PG-11 CFS was mixed with 10 ml of the prepared *S*. Typhimurium suspension. The cell growth of *S*. Typhimurium ATCC 14028 was monitored every two hours for a total duration of 24 h. Fresh MRS broth with the same treatment was used as a control.

### PI Uptake Analysis

To demonstrate the effect of *L. brevis* PG-11 CFS on the integrity of *S*. Typhimurium ATCC 14028 cells, the PI uptake assay was performed according to Crowley *et al*. [39] and Yi *et al*. [40] reported with minor modifications. The activated *S*. Typhimurium ATCC 14028 was centrifuged at 7,378 ×*g* and 4°C for 5 min, washed thrice with PBS (Servicebio, China, 0.01 mol/l pH 7.5), suspended in LB broth, and adjusted to 10^6^ CFU/ml for use. Subsequently, the suspension was treated with *L. brevis* PG-11 CFS (10 ml) for 0.5, 1 and 2 h, then centrifuged and washed with PBS; the same treatment with PBS was used as a control. As described by the manufacturer, the samples were incubated with PI (2 mg/ml, Sangon) at 37°C for 20 min in the dark. After incubation, the treated cells were photographed using a fluorescence microscope.

### Determination of Intracellular ATP

To determine the action of *L. brevis* PG-11 CFS on *S*. Typhimurium ATCC 14028 cells, the intracellular ATP concentrations were measured as described by Rao at *et al*. [41] and Liang *et al*. [42], with slight modifications. The exponential growth phase cultures of *S*. Typhimurium ATCC 14028 were centrifuged at 7,378 ×*g* and 4°C for 5 min and the supernatants were removed. The cell pellet was washed three times with PBS, collected by centrifugation, and then suspended in LB broth. The equal volume suspension was treated with *L. brevis* PG-11 CFS (10 ml) for 0.5, 1 and 2 h respectively. Subsequently, the precipitate was collected by centrifugation, washed three times with PBS, and the supernatant was removed. According to the instructions provided by the supplier (Solarbio, China), the ATP level in the cells was detected using an ATP assay kit.

### SEM Analysis

To investigate the damage to *S*. Typhimurium ATCC 14028 cells with CFS, the method was described by Wang *et al*. (2019) [43]. Exponential growth phase *S*. Typhimurium ATCC 14028 suspension (OD_600_ =0.6) was centrifuged 7,378 g 4°C for 5 min and washed three times with PBS. The *L. brevis* PG-11 CFS (10 ml) was mixed with an equal volume of *S*. Typhimurium ATCC 14028 suspension and incubated at 37°C for 2 h. PBS (0.1 mol/l pH 7.4) was used as a control in the same way. Subsequently, *S*. Typhimurium ATCC 14028 cells were harvested and fixed with 2.5% glutaraldehyde (Sangon) at 4°C overnight and dehydrated using a series of gradient ethanol solutions (30, 50, 70, 80, 90, and 100%) at 4°C dehydrated. After natural drying, the cells were placed on a slide, coated with gold, and the morphology was observed using SEM.

### Statistical Analysis

The data were repeated three times and the final results are taken as mean ± standard deviation. Analysis of statistical significance was performed with one-way analysis of variance using IBM SPSS Statistics 22.0. The post hoc testing used Duncan's multiple range test and statistical differences were described as *P* values ≤ 0.05.

## Results

### Evaluation of Antimicrobial Activity Analysis

After adjusting the pH of CFS, the effects of organic acid were excluded, and the CFS, with the addition of catalase, still exhibited antimicrobial activity, suggesting that *L. brevis* PG-11 may produce bacteriocin. The CFS of *L. brevis* PG-11 antibacterial spectrum and MIC are presented in Table 1. The antimicrobial activity of CFS (1 × MIC) and Nisin Z were observed against all indicator bacteria. The CFS exhibited a range of antimicrobial activities against both Gram-negative and Gram-positive bacteria, including *S*. Typhimurium, *E. coli*, *K. pneumoniae*, *A. baumannii*, *P. aeruginosa*, *S. aureus* and *S. epidermidis*. Similar to the characteristics of Nisin Z, it is speculated that CFS may be a type of broad-spectrum BLIS.

### The Analytics of BLIS Molecular Weight Size

**Fig. 1:**
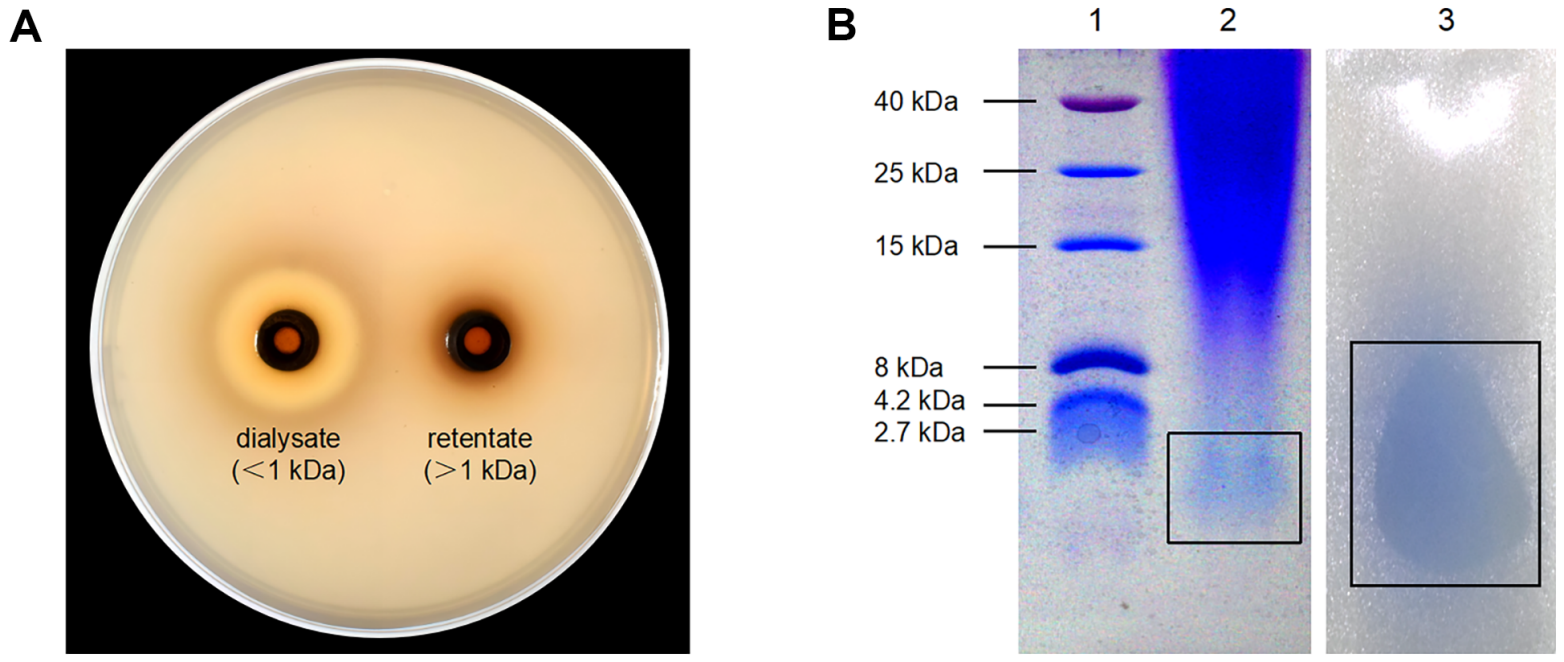
Molecular weight determination of BLIS produced by *L. brevis* PG-11 (A) Antimicrobial activity assay of dialysate and retentate. (**B1**) The protein bands in the first lane on polyacrylamide gel showed an ultra-low molecular weight rainbow protein marker. (**B2**) The second lane showed a single protein of *L. brevis* PG-11 bacteriocin-like band with a low molecular weight. (**B3**) Another part of the gel used for the antibacterial experiment showed an inhibition zone on the covered culture medium plate.

As shown in Fig. 1A, the crude bacteriocin obtained by dialysis (component <1000 Da) exhibited an inhibition zone diameter of 20.1 ± 0.2 mm against *S*. Typhimurium ATCC 14028. Active dialysate obtained from the concentrate was analyzed by 18% acrylamide Tricine-SDS-PAGE. As shown in Fig. 1B, the extracted crude bacteriocin of *L. brevis* PG-11 showed a low molecular mass band below 2.7 kDa stained with Coomassie brilliant blue stained. A clear inhibition area appeared at the location of the band, indicating that the BLIS from *L. brevis* PG-11 was a low molecular weight bacteriocin-like substance.

### Effect of pH, Heat, Proteases and UV Rays on the Antibacterial Activity

**Fig. 2:**
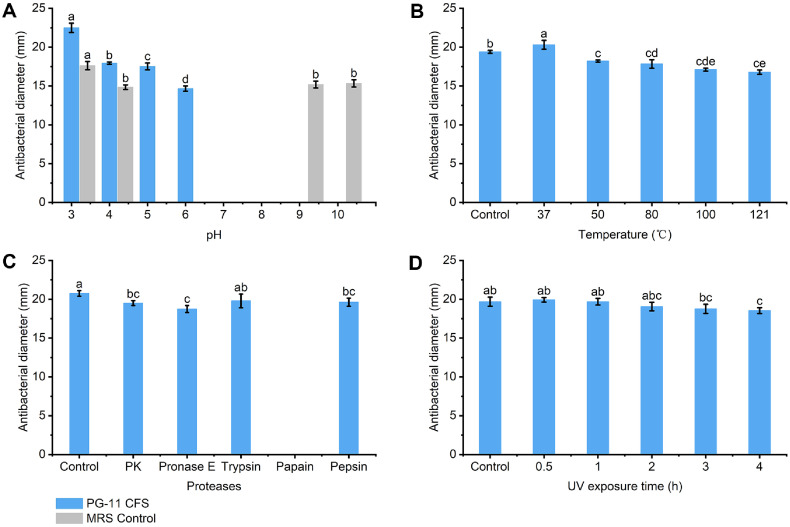
Antimicrobial characteristics of BLIS produced by *L. brevis* PG-11 as affected by pH, temperature, proteases, UV exposure. (**A**) The pH of PG-11 CFS and MRS broth was adjusted to the same and then their antibacterial diameter was *measured*; (**B**) The antibacterial activity of PG-11 CFS treated by different temperatures; (**C**) Residual antibacterial activity of PG-11 CFS under different protease treatments; (**D**) Effect of duration of UV exposure on PG-11 CFS antimicrobial activity. The significant differences (*p* ≤ 0.05) between each parameter defined different letters in the same figure.

As shown in Figure 2A, the pH stability of CFS was examined. The pH treatment significantly affected the antibacterial effect of PG-11 CFS. During treatment at different pH levels, as the pH increased, a distinct decline in the antimicrobial activity of CFS was observed. Similar to other LAB bacteriocins, PG-11 CFS maintained antimicrobial activity at pH 3.0-6.0. In Figure 2B, the CFS of *L. brevis* PG-11 maintained between 86.5% and 93.9%after heat treatment. Although antimicrobial activity was affected by increased temperatures, maximum activity occurred at 37°C. The antimicrobial activity of CFS of *L. brevis* PG-11 showed high thermal stability when heated at 100°C for 1 h and 121°C for 20 min (autoclave) during heat treatment. The antimicrobial activity of *L. brevis* PG-11 CFS was highly sensitive to papain, as shown in Fig. 2C. However, only slight effects were observed from treatment with proteinase K, pronase E, pepsin, and trypsin on the CFS activity of *L. brevis* PG-11. The results showed that the antimicrobial substances in PG-11 CFS are protease-sensitive, suggesting that they are a BLIS. As shown in Figure 2D, the BLIS produced by *L. brevis* PG-11 maintained complete stability after 0.5 h and 1h of UV exposure and partial stability after exposure for 2, 3, and 4 h. The slight decrease in antimicrobial activity indicated that the BLIS was highly resistant to UV radiation.

### The BLIS Production Kinetics

**Fig. 3:**
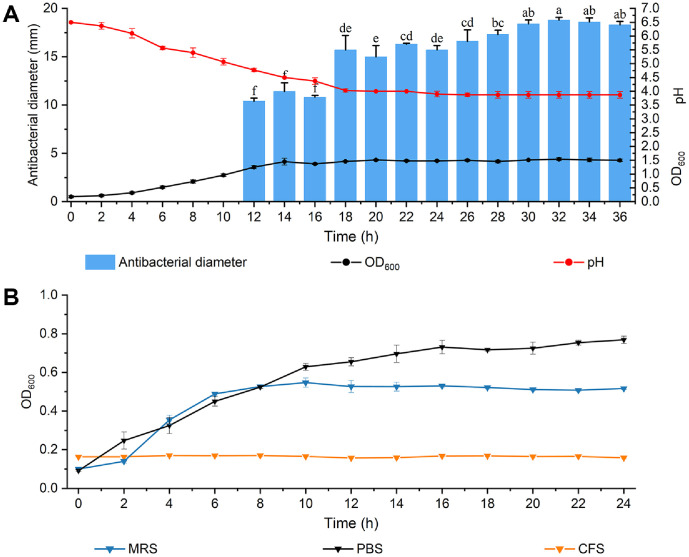
BLIS produced by *L. brevis* PG-11 synthesis kinetics and the growth of *S*. Typhimurium ATCC 14028. (**A**) Relationship between antimicrobial activity and incubation time and pH changes in the culture solution; (**B**) PBS, MRS broth and PG-11 CFS on the growth of *S*. Typhimurium ATCC 14028. The significant difference (*p* ≤ 0.05) was indicated by different letters between each value.

The production of BLIS from *L. brevis* PG-11 in MRS broth is shown in Fig. 3A. Extracellular inhibitory activity was first detectable after 12 h of growth during the late exponential growth phase. The inhibition zone diameter was 10.4 ± 0.32 mm. As the incubation time increases, the synthesis and secretion of BLIS activity level constantly improves, and the inhibition zone of CFS was maximally produced at 32 h, reaching 18.8 ± 0.3 mm. Antimicrobial activity stopped increasing during the growth plateau, followed by a slight decrease in activity during this phase. The pH value of the culture dropped from 6.5 to 3.9, and the pH became stable after 24 h of culture. This shows that *L. brevis* PG-11 has strong acid resistance and the BLIS produced is adapted to the acidic environment.

### The Growth Curves of *S*. Typhimurium ATCC 14028

There was a large increase during the first 2 h for control group shown in Figure 3B. Compared to PBS, the bacterial growth in the mixture of MRS broth and *S*. Typhimurium ATCC 14028 was partially inhibited. Inhibition was observed in the MRS broth. This may be due to the presence of natural inhibitory components in the culture medium against *S*. Typhimurium ATCC 14028. However, the more significant inhibition suggests that the addition of BLIS caused rapid growth arrest in *S*. Typhimurium ATCC 14028. These results suggest that *L. brevis* PG-11 BLIS can disrupt the normal growth cycle of bacteria and thus fight against *S*. Typhimurium ATCC 14028.

### Fluorescence Imaging with PI Uptake

**Fig. 4:**
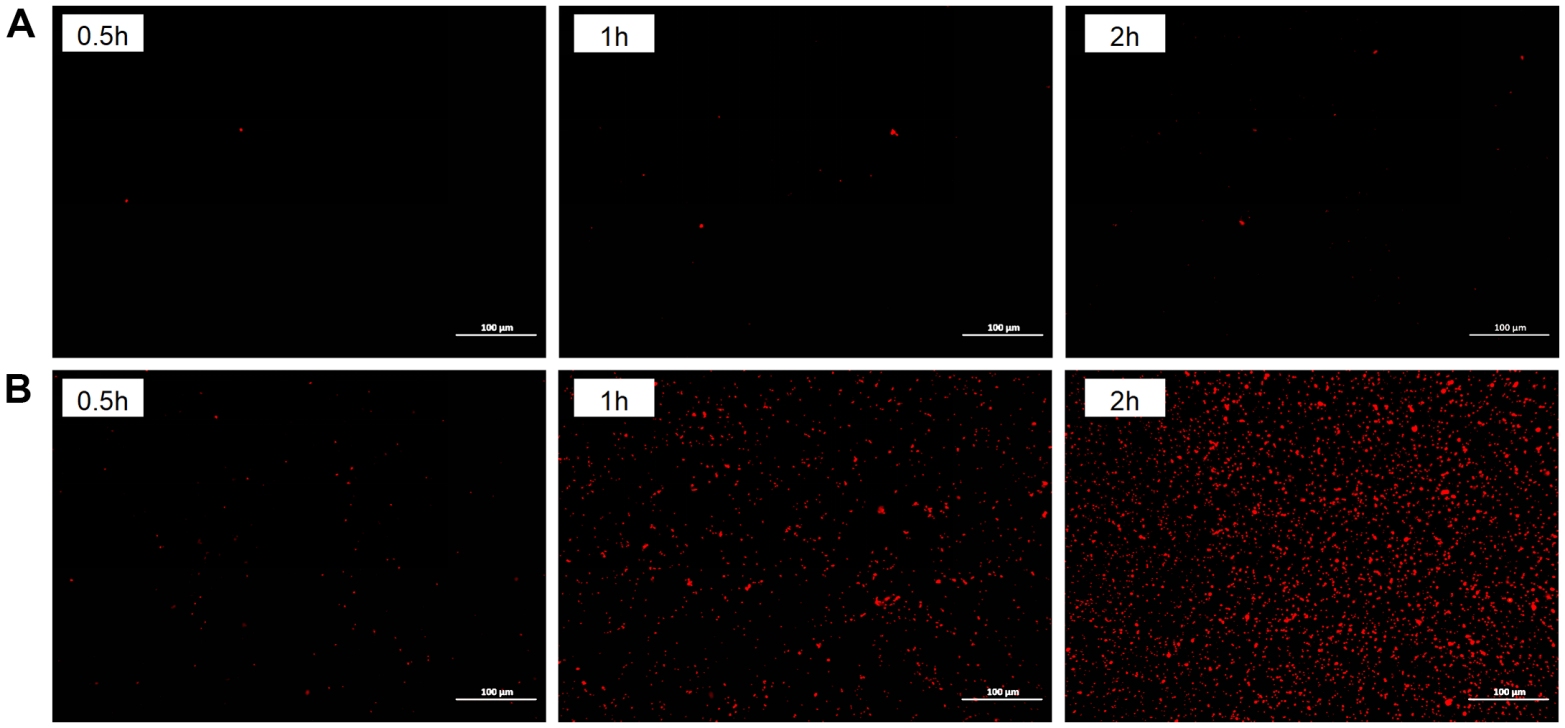
The effect of BLIS on the cell membrane integrity of the *S*. Typhimurium ATCC 14028. (**A**) The PBS was used as a control that treated *S*. Typhimurium ATCC 14028 for 0.5, 1 and 2 h; (**B**) Area of fluorescence shown by BLIS from PG- 11 acted on the *S*. Typhimurium ATCC 14028 cells for 0.5, 1 and 2h, respectively.

The effects of *L. brevis* PG-11 BLIS on the cell morphology of *S*. Typhimurium ATCC 14028 were visualized using fluorescence microscopy, as shown in Figure 4. PI, a small fluorescent molecule, selectively stains dead cells by emitting red fluorescence when it penetrates compromised cell membranes and binds to nucleic acids [39]. Compared to the untreated control group, red fluorescence was observed in the BLIS-treated samples, indicating cell membrane damage. The intensity of red fluorescence increased with the duration of BLIS treatment. As shown in Figure 4B, *S*. Typhimurium ATCC 14028 cells exhibited low fluorescence intensity after 0.5 h of treatment. After 1 h, a slight increase in fluorescence intensity was detected. When the incubation period was extended to 2 h, a more extensive fluorescence signal was observed, suggesting significant membrane disruption. These observations suggest that BLIS treatment disrupts bacterial cell membrane integrity, leading to nucleic acid leakage. Simultaneously, the dye penetrated the cells and bind to the DNA.

### Intracellular ATP Leakage

**Fig. 5:**
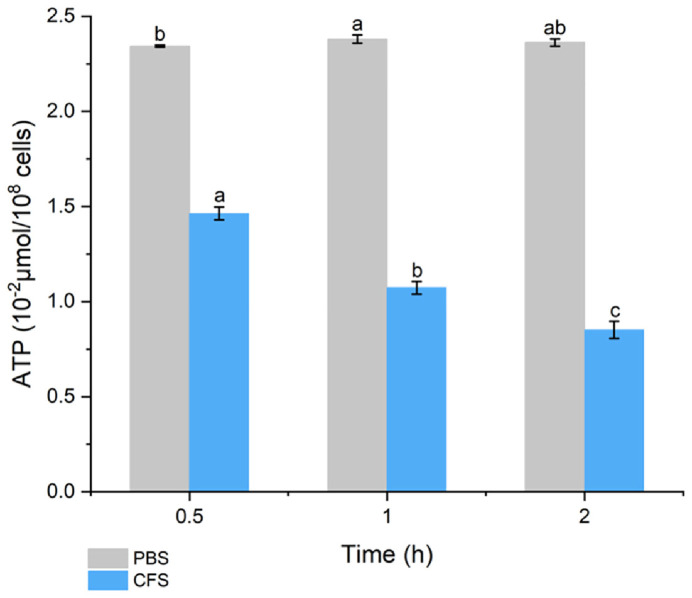
Effects of BLIS incubation for different time periods on ATP concentration in *S*. Typhimurium ATCC 14028 cells. The significant difference (*p* ≤ 0.05) was indicated by different letters between the each parameter.

The results are shown in Figure 5. After BLIS treatment, the intracellular ATP concentration decreased, indicating that BLIS may have destroyed the integrity of the *S*. Typhimurium ATCC 14028 cells. At 0.5 h, the ATP concentration in the control group was 0.234 μmol/10^8^ cells, and remained almost unchanged with the extension of incubation time. The ATP concentration was 0.146 μmol/10^8^ after 0.5 h of BLIS treatment and continued to decrease with the extension of incubation time. It is evident that the BLIS produced by *L. brevis* PG-11 changes the cell membrane permeability of *S*. Typhimurium ATCC 14028, leading to ATP leakage.

### Observation of the Morphology of *S*. Typhimurium Cells Using SEM

**Fig. 6:**
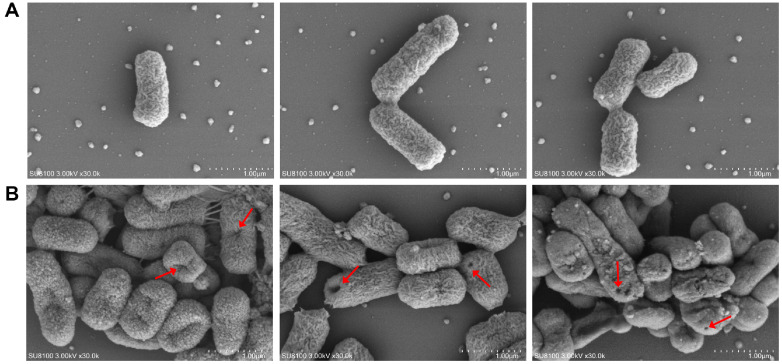
The SEM assay of *S*. Typhimurium ATCC 14028 cell treated with BLIS from *L. brevis* PG-11corroborate the damage results. (**A**) The ultramorphology of *S*. Typhimurium ATCC 14028 treated by PBS 1 h; (**B**) *S*. Typhimurium ATCC 14028 showed pore formation, structural collapse and deformation appeared after 1h of BLIS treatment. The figure shown in the results are for different photo angles of the same sample.

The effects of *L. brevis* PG-11 BLIS on the morphology of *S*. Typhimurium ATCC 14028 cells were evaluated through scanning electron microscopy, as shown in Figure 6. Morphological changes were observed after 1 h of BLIS treatment. In the control group in Fig. 6A, *S*. Typhimurium ATCC 14028 cells exhibited intact cell structures with smooth surfaces and typical rod-shaped profiles, characteristic of healthy bacterial cells. As shown in Figure 6B, significant morphological alterations were observed in the BLIS-treated cells. These cells showed wrinkling, collapse, and expanded cellular surface, along with the formation of hollow regions. Furthermore, extensive deformation and distortion of the cells were evident, likely caused by BLIS-induced damage to the cell membrane. These findings suggest that the BLIS produced by *L. brevis* PG-11 targets the bacterial membrane, leading to structural destabilization and cell death.

## Discussion

In this study, BLIS produced by *L. brevis* PG-11 showed strong antibacterial activity against *S*. Typhimurium ATCC 14028, which was similar to that of L.brevis DF01 isolated from a sample of kimchi as reported in previous studies [21, 44]. The BLIS produced by L.brevis PG-11 demonstrated broad-spectrum activity against several foodborne and human pathogens, including *E. coli*, *S. aureus*, *K. pneumoniae*, *A. baumannii* and *S. epidermidis*. The minimum inhibitory concentration (MIC) of BLIS against standard strains of *E. coli* ATCC 25922, *S*. Typhimurium ATCC 14028, and *S. aureus* ATCC 25923 was 62.5, 125, and 125 mg/ml. The MIC values for clinically isolated *S*. Typhimurium SH107, *K. pneumoniae* TY-30, *A. baumannii* ZXL-113, *P. aeruginosa* Z05, and *S. epidermidis* MR-75 are 62.5, 125, 62.5, 62.5, 125 mg/ml. Similar results were observed in the study by [28]. This suggests that the BLIS has significant potential as a biopreservative for food storage or as a component in antimicrobial food packaging. Low molecular weight bacteriocins produced by *L. brevis* strains have been reported previously. The apparent molecular weight of bacteriocin of *L. brevis* KLDS1.0373 isolated from traditional Chinese fermented cream is 3.8 kDa [45], the anti-*Helicobacter pylori* bacteriocin produced by *L. brevis* BK11 was estimated to have a molecular weight of approximately 6.5 kDa [46]. The molecular weight of BLIS of *L. brevis* PG-11 is significantly lower than that of most *L. brevis* bacteriocins reported so far, and an ultra-low molecular weight of < 1 kDa is rare among *L. brevis* bacteriocins and is rarely reported even in LAB. Similar low-molecular-weight bacteriocins (less than 1 kDa) have been identified in other LAB, such as the hexapeptide LNFLKK from *Lactiplantibacillus plantarum* NMGL2 (761.95 Da) and Plantaricin GZ1-27 (975 Da) [36, 47]. Beyond LAB, antimicrobial peptides from *Bacillus subtilis* LFB112, with molecular weights of 400-800 Da, also demonstrate high antibacterial potency against diverse pathogens [48].

For use in food preservation, bacteriocins must withstand harsh conditions, including low pH and high temperatures [27]. The BLIS from PG-11 exhibited remarkable stability under acidic conditions, consistent with findings from Peng *et al*. [49], Zhang *et al*. (2022) [50], and Han *et al*. [51]. LAB bacteriocins generally show enhanced stability at low pH, potentially due to the fact that the solubility increased at lower pH values (pH ≤ 5.0) and there are more interactions between the target cells and bacteriocin [50]. Conversely, in alkaline environments, protein denaturation may occur due to the separation of amino and carboxyl groups [52]. Heat stability was also observed, with antibacterial activity of BLIS produced by PG-11 retained after treatment at 121°C for 20 min, similar to other low-molecular-weight LAB bacteriocins [53-55]. Such thermal stability is likely associated with the small, globular structures, hydrophobic regions, stable cross-linkages, and high glycine content of the peptides [36, 56]. The BLIS from PG-11 was completely inactivated by papain but not by other proteases, indicating specificity to certain hydrophobic amino acid residues that serve as cleavage sites [57]. This specificity was similarly observed in bacteriocins from *Lacticaseibacillus paracasei* Zhang [58]. Papain, a cysteine protease, has a preference for cleavage on peptide bonds of basic amino acids, especially the residues following arginine, lysine and phenylalanine [59]. Pepsin, trypsin, proteinase K, and pronase E are different. There were found that pepsin preferentially cleaves the site of peptides that contain aromatic or leucine residues; trypsin specifically cleaves lysine and arginine residues unless the connection is proline; proteinase K has a preference for cleavage on the carboxyl side of specific amino acids and denoted as a non-specific proteinase; pronase E selectively prefers to hydrolyze peptide bonds formed by glutamic acid or aspartic acid carboxyl groups [60-64]. Prolonged UV exposure could alter the protein structure and function. Hassan *et al*. (2020) [65] reported that the bacteriocin of *Lactobacillus spiralis* had a weakened antagonistic activity against indicator bacteria after 30 min of ultraviolet irradiation. In our study, UV irradiation did not affect the activity of BLIS of PG-11 within 1 h, but exposure to 4 h slightly reduced the activity of BLIS. PG-11 BLIS showed excellent UV resistance, and long-term exposure did not cause it to lose its antibacterial activity, which is similar to the results reported by A.I and A [66]. Maximum antibacterial activity was detected after 32 h of culture, corresponding to a pH reduction from 6.5 to 3.9, which stabilized after 24 h. In most bacteriocins, the lower pH (≤5) could stabilize the antibacterial activity [36]. The stabilisation level of BLIS in the stationary phase indicated that it could be a secondary metabolite. This result is consistent with other studies [36, 38].

To further demonstrate the mode of action of BLIS from PG-11, the inhibited growth curve of *S*. Typhimurium ATCC 14028, PI uptake and SEM assay were examined. Our observations indicate that the growth of *S*. Typhimurium ATCC 14028 was obstructed significantly by BLIS from *L. brevis* PG-11. One key mechanism by which the bacteriocin inhibits Gram-negative bacteria is by interfering with nucleic acid and protein metabolism, thereby inhibiting growth [17, 67]. Compared to controls, the absorbance was stable long after BLIS treatment, suggesting that BLIS completely altered the normal growth of *S*. Typhimurium ATCC 14028. Similar results were reported [51, 55, 68]. The PI uptake assay exhibited that BLIS disrupted intact cell membrane leading to DNA leakage and this is related to the treatment time. Since PI binds to DNA, the fluorescence density of the cells changed as the cells died, and high fluorescence was shown upon the action of BLIS after 2 h. Similar results were also reported by Zhang *et al*. (2022) [50] who found that bacteriocins disrupted the integrity of target bacterial membranes. The intracellular ATP concentration test showed that the ATP concentration of *Salmonella* typhimurium cells treated with BLIS was significantly lower than that of the control group, and showed a time-dependent relationship. Similar results were obtained in the report of Rao *et al*. [40], where the metabolites of *L. plantarum* reduced the intracellular ATP level of *Pseudomonas lundensis* in a concentration-dependent manner. The decrease in intracellular ATP levels is due to the increased permeability of the cell membrane, which leads to the leakage of ATP; or BLIS reduces the activity of ATPase and inhibits the normal activity of cells, thereby achieving an antibacterial effect [41, 69]. This result further confirms the effect of BLIS on the integrity of *S*. Typhimurium ATCC 14028 cell membranes. In addition, SEM analysis confirmed that when PG-11 BLIS attacked the cell membrane of *S*. Typhimurium ATCC 14028, the cell surface morphology changed significantly, wrinkled, deformed, and formed clear pores. The CFS of *L. brevis* Lb13H acts on *Listeria monocytogenes* cells, causing wrinkles, dents, ruptures in the cell wall, and folding of the cell, but no pores are formed [28]. The cell surface is wrinkled and the cell is flattened or leaking air as a whole. The distortion and shrinkage may be caused by increased cell membrane permeability and leakage of small molecules [70]. Bacteriocins cause bacterial death primarily by forming pores or ion channels in the cell membrane [71, 72]. The pore formation could be mediated by the hydrophobic part of bacteriocin with the positively charged, integration with phosphate group of negatively charged in the cell membrane electrostatic interactions, thus resulting in leakage of cellular materials to effectively kill the target cell [15, 73]. Based on this, we speculated that one of the mode of action of BLIS from *L. brevis* PG-11 could disrupt the cell membrane of *S*. Typhimurium ATCC 14028 and perforate pores, leading to cell morphology changes and leakage of contents, inhibiting the metabolism of grow. Similarly, this damage to target cells caused by LAB bacteriocin was discovered by other researchers [40, 43, 52, 74].

## Conclusion

The BLIS produced by *L. brevis* PG-11 as a bacteriocin-like inhibitory substances with a molecular weight less than 1 kDa which has effective and stable antimicrobial activity against *S*. Typhimurium ATCC 14028. Further investigation into the antimicrobial mechanism shows that BLIS could damage the integrity of the cell membrane, leading to leakage of nucleic acid and ATP and attack the membrane to cause a distinct pore formation, ultimately leading to cell death. Therefore, the BLIS produced by *L. brevis* PG-11 could be a potential and natural antibacterial agent for protection against *S*. Typhimurium infections in various food systems. However, further studies involving purification identification and structural analysis of BLIS are needed and will be the subject of our future work.

## Figures and Tables

**Table 1:** The antimicrobial spectrum and MIC of CFS produced by PG-11.

Indicator strain	Source	Gram	CFS MIC value (mg/ml)	Nisin Z MIC value (mg/ml)
*S.* Typhimurium	ATCC 14028	G-	62.50	0.977
*S*. Typhimurium	SH107	G-	62.50	0.977
*E. coli*	ATCC 25922	G-	125.0	1.953
*K. pneumoniae*	TY-30	G-	125.0	1.953
*A. baumannii*	ZXL-113	G-	62.50	0.488
*P. aeruginosa*	Z05	G-	62.50	0.488
*S. aureus*	ATCC 25923	G+	125.0	0.015
*S. epidermidis*	MR-75	G+	125.0	0.015
